# Effects of procedural justice on prospective antidepressant medication prescription: a longitudinal study on Swedish workers

**DOI:** 10.1186/s12889-020-08560-5

**Published:** 2020-04-15

**Authors:** Viktor Persson, Constanze Eib, Claudia Bernhard-Oettel, Constanze Leineweber

**Affiliations:** 1 Stress Research Institute, Stockholm University, Stockholm, Sweden; 2 Department of Psychology, Uppsala University, Uppsala, Sweden; 3 Department of Psychology, Stockholm University, Stockholm, Sweden

**Keywords:** Organizational justice, Procedural justice, Register data, Antidepressant medication, Prescription medicine

## Abstract

**Background:**

Procedural justice has been linked to several mental health problems, but most studies have used self-reported data. There exist a need to assess the link between procedural justice and health using outcomes that are not only self-reported. The aim of the current study was to examine whether perceived procedural justice at work is prospectively associated with antidepressant medication prescription.

**Methods:**

Data from 4374 participants from the Swedish Longitudinal Survey of Health (SLOSH) were linked to the Swedish National Prescribed Drug register. Based on their perceived procedural justice at two times (2010 and 2012), participants were divided into four groups: stable low, increasing, decreasing and stable high justice perceptions. Using Cox regression, we studied how the course of stability and change in perceived procedural justice affected the rate of prescription of antidepressant medication over the next 2 years. Participants with missing data and those who had been prescribed antidepressant medication in the period leading up to 2012 were excluded in the main analyses to determine incident morbidity.

**Results:**

The results showed that after adjustment for sex, age, education, socioeconomic position, marital status, and insecure employment a decrease in perceived procedural justice over time was associated with greater receipt of antidepressants compared to people with stable high perceptions of procedural justice (HR 1.76, 95% CI: 1.16 to 2.68). Being female and having insecure employment were also associated with higher hazards of antidepressant prescription.

**Conclusions:**

These findings strengthen the notion that procedural justice at work influences psychological well-being, as well as provide new insights into how procedural justice perceptions may affect mental health.

## Background

According to the World Health Organization (WHO), depression is a common and serious health problem globally, with more than 264 million people affected [1, 2]. The point prevalence of major depressive disorder in 2009 in Sweden was 5.2%, while 10.8% of the population had a clinically significant ongoing depression [3]. According to the 2018 national public health survey in Sweden, 4.0% of the population had been diagnosed with depression from a doctor in the past year, with an additional 14.0% who had been diagnosed in prior years [4]. Generally, women are twice as likely to be affected by depression as men [3, 5, 6]. While the etiology of depression is multifaceted, psychosocial working conditions, major life events, individual personality, substance abuse as well as genetics all have been linked to depression onset [5, 7–14]. A review by Bonde published in 2008 reported strong links between psychosocial working conditions and subsequent depressive symptoms but concluded that, due to methodological issues (e.g., common method bias), conclusions regarding causal inference could not be made. Systematic reviews provided additional evidence for the relationship between psychosocial working conditions and depression [12, 14], but also highlighted the need for studies to examine exposures that are measured independently from the outcomes.

One important aspect of the psychosocial work environment is organizational justice, which is commonly divided into three components: distributive justice, which regards the perceived fairness of resource allocations, interactional justice, which concerns the treatment of employees by supervisors, and procedural justice, which relates to the formal decision-making in the organization [15]. Procedural justice is one of the most studied justice dimensions and has been shown to have substantial relations to health outcomes [16, 17]. Procedural justice provides employees with a sense of predictability, makes it more likely to secure future positive outcomes, and facilitates a sense of identification and perceptions of self-esteem [18]. Some researchers have suggested the allostatic load model as the causal pathway from low procedural justice to ill-health [19]. The allostatic load model posits that the body responds to a stressor by physiological adaptation, such as getting ready for ‘fight or flight’. This is beneficial in the short run, but with a prolonged stressor, the allostatic load becomes maladaptive, causing harm to the physiological system. This, in turn, can cause a multitude of different health problems, such as heightened blood pressure, insomnia and depression [20, 21]. Multiple studies have shown links between low perceived procedural justice and diverse health measures, including depression [7, 10, 19, 22–25]. Despite the evidence that organizational justice is related to depressive states, weaknesses in the existing empirical evidence remain. Most research relied on cross-sectional designs, which limits claims for causality [7, 14, 26, 27]. Only very few studies have investigated change in justice perceptions over time, and how this affected subsequent health [28–30]. A recent study by Eib et al. (2018), demonstrated longitudinal effects from procedural justice to depressive symptoms as well as reversed effects, that is, depressive symptoms predicted subsequent procedural justice [19]. In another study by Åhlin et al. (2019), high procedural justice was associated with lower self-reported depressive symptoms at the point of measurement, but no effect in neither direction could be seen 2 years later [31]. Where scholars have employed longitudinal designs, they mostly used self-report measures for both exposures and outcomes, an approach which may generate common method bias (see e.g., Theorell et al., 2015; Madsen et al., 2018, for comprehensive overviews) [12, 14]. In an effort to address these shortcomings, Grynderup et al. (2012) found that procedural justice predicted onset of depression, measured with a psychiatric diagnostic interview, over a follow-up time of 2 years.

Taken together, more work is needed for well-founded conclusions about causality in the procedural justice–depression relationship. This study addresses two shortcomings in past research: the use of self-reported measures of exposure and outcome, and the measurement of exposure and outcome at the same time. Firstly, to circumvent self-reported health data this study focuses on antidepressant medication prescription, which can also serve as indicator for a variety of mental health disorders. The focus on medication has the advantage that it is an objective health indicator. Many studies have shown that objective health indicators, e.g. medication prescription, are a reliable and valid alternative to self-reported health measures. For example, a Swedish study by Henriksson et al. (2003) did show that 82% of those receiving SSRI’s also had a diagnosis of depression (see also Gardarsdottir et al., 2007) [32, 33]. Despite the fact that antidepressant medication does not necessarily equal depression, there are examples of studies using antidepressant medication as a proxy for depression [34, 35]. The advantage of using prescribed medication over self-reported data is that the risk of subjective bias is eliminated. Secondly, we employ a prospective design, which enables us to investigate how changes in procedural justice relate to medication prescription. Changes in procedural justice over time have rarely been studied in the justice literature [28, 30] and this study therefore investigates how changes of procedural justice relate to the onset and continuous receipt of antidepressant medication. The use of a prospective design in combination with objective and independently measured indicators of mental health disorders, such as antidepressant medication prescription from official registers, could provide one further step towards causal inference.

Hence, this paper poses the question whether perceived procedural justice at work and changes in the very same over two time points separated by 2 years are associated with prescribed antidepressant medicine during a follow-up period of approximately two and a half year. We hypothesized that (1) stable low procedural justice, as well as (2) a change from high to low perceived procedural justice over time, would result in a higher prevalence of prescriptions of antidepressant medication, compared to high procedural justice over time. Further, we expect that (3) a change from low to high procedural justice does not associate with a higher prevalence of antidepressant medication as compared to those who report high procedural justice.

## Methods

### Participants

We analyzed data from the SLOSH (Swedish Longitudinal Occupational Survey of Health) study. SLOSH is a repeated biennial cohort survey, which focuses on work environment and health. The first data collection was conducted in 2006. It has since been expanded with more respondents from the Swedish Work Environment Surveys (SWES), and today the SLOSH sample includes all eligible respondents to SWES 2003–2011 (*n* = 40,877). SLOSH consists of a questionnaire in two versions, one for those currently in paid work and one for those working below 30% of full-time, or who are permanently or temporarily outside the labor force. Response rates varied between 65% in 2006 and 48% in 2018. For further details see elsewhere [36]. The study sample used for the current study includes participants who were gainfully employed for at least 30% of full-time in both 2010 and 2012 (*n* = 5569) and answered all questions about procedural justice at the workplace in both the data collection of 2010 and 2012 (*n* = 5056) and did not have a prescription of antidepressant medication during the 2 years prior to answering SLOSH in 2012 (*n* = 4756). Finally, anyone with missing data on any of the covariates were excluded, and our final sample included 4374 individuals. Those with missing data on one or more variables were on average more likely to be older, male, and lower educated (*p* < 0.01). Both SLOSH as well as the present study have been approved by the Regional Research Ethics Board in Stockholm. All participants provided their informed consent.

### Measures

*Procedural justice perceptions* were measured with a 7-item scale adopted from Moorman (α = .91 for both 2010 and 2012) [37]. The items reflect whether decision-making procedures at the workplace are accurate, correctable, consistently applied, and whether the procedures include opinions from the people involved. Responses ranged from 1 (*totally agree*) to 5 (*totally disagree*). Each item was then reversed so that a higher score reflects a more positive perception of procedural justice. For analysis, four groups were constructed. First, we created a sum score for each participant ranging from 7 to 35 for each wave. Then, we split the scores of procedural justice at 2010 and 2012 at the lower quartile (19.0 in both 2010 and 2012). Such, we got two groups, one with high perceived procedural justice (75% of the sample) and one with low perceived procedural justice (remaining 25% of the sample). Finally, these two (justice 2010) x two (justice 2012) groups were combined to create a total of four groups: *‘Stable low’* (low perceived justice in both 2010 and 2012, *n* = 645), *‘Increasing’* (low perceived justice in 2010, high perceived justice in 2012, *n* = 546), ‘*Decreasing’* (high perceived justice in 2010, low perceived justice in 2012, *n* = 535) and *‘Stable high’* (high perceived justice in 2010 and 2012, *n* = 2648).

The exposure variable was *antidepressant medication prescriptions*. Information on prescribed antidepressants were obtained from the Swedish National Prescribed Drug register, which contains data for all prescriptions collected from Swedish pharmacies. All medication classified as N06A-drugs in the Anatomical Therapeutic Chemical classification system were defined as antidepressant medication, including selective serotonin reuptake inhibitors (SSRI) and other antidepressant medication. Individuals were followed from the exact date of answering SLOSH in 2012 (varying from April, 24, 2012 until October, 4, 2012) until first time of antidepressants prescription or December, 31, 2014 (the latest date available), whichever came first. Main analyses excluded participants with any ongoing or previous prescription of antidepressants during the 2 years prior to answering SLOSH 2012 (*n* = 278).

*Covariates* were chosen using the theory-driven approach of directed acyclic graphs. Sex, age, socioeconomic position, marital status, insecure job situation and educational level in 2010 were included as covariates, see Table 1 for an overview. All covariates were taken from register data except for insecure job situation which was measured with the question “*What type of employment do you have?*” in 2010. Individuals who answered that they were employed in projects, as substitutes or on an hourly basis were defined as having an insecure job situation.

**Table 1:** Sample characteristics (*n* = 4374)

Demographics	Group 1: Stable low (*n* = 645)	Group 2: Increasing (*n* = 546)	Group 3: Decreasing (*n* = 535)	Group 4: Stable high (*n* = 2648)	Total (*n* = 4374)	*p*-value
Mean justice in 2010 (SD)	14.9 (3.2)	15.8 (2.9)	24.0 (3.5)	26.4 (4.2)	23.1 (6.2)	*p* < 0.001
Mean justice in 2012 (SD)	14.6 (3.2)	24.5 (3.7)	16.0 (2.8)	26.5 (4.3)	23.2 (6.3)	*p* < 0.001
*Sex*						
Female	389 (60.3)	308 (56.4)	292 (54.6)	1480 (55.9)	2469 (56.4)	
Male	256 (39.7)	238 (43.6)	243 (45.4)	1168 (44.1)	1905 (43.6)	
Mean age in 2010 (SD)	48.5 (9.1)	48.9 (9.0)	47.8 (9.3)	49.8 (9.3)	49.3 (9.3)	
*Age*						*p* < 0.001
22–39 years	116 (18.1)	95 (17.4)	122 (22.8)	424 (16.0)	757 (17.3)	
40–49 years	216 (33.5)	170 (31.1)	167 (31.2)	757 (28.6)	1310 (29.9)	
50–59 years	234 (36.3)	217 (39.7)	183 (34.2)	1029 (38.8)	1663 (38.0)	
60–71 years	79 (12.2)	64 (11.7)	63 (11.8)	438 (16.5)	644 (14.7)	
*Education in 2010*						*p* < 0.001
Compulsory	57 (8.8)	53 (9.7)	48 (9.0)	320 (12.1)	478 (10.9)	
2-year upper secondary	134 (20.8)	126 (23.1)	106 (19.8)	645 (24.4)	1011 (23.1)	
3 or 4-year upper secondary	146 (22.6)	117 (21.4)	110 (20.6)	618 (23.3)	991 (22.7)	
University or equivalent shorter than 3 years	105 (16.3)	86 (15.8)	88 (16.5)	348 (13.1)	627 (14.3)	
University or equivalent 3 years or longer	203 (31.5)	164 (30.0)	183 (34.2)	717 (27.1)	1267 (29.0)	
*Marital status in 2010*						
Married	335 (51.9)	319 (58.4)	299 (55.9)	1567 (59.2)	2520 (57.6)	
Unmarried	216 (33.5)	160 (29.3)	164 (30.7)	722 (27.3)	1262 (28.9)	
Divorced	88 (13.6)	60 (11.0)	68 (12.7)	325 (12.3)	541 (12.4)	
Widowed	6 (0.9)	7 (1.3)	4 (0.8)	34 (1.3)	51 (1.2)	
*Employment security in 2010*						
Secure	627 (97.2)	528 (96.7)	511 (95.5)	2542 (96.0)	4208 (96.2)	
Insecure	18 (2.8)	18 (3.3)	24 (4.5)	106 (4.0)	166 (3.8)	
*Number (%) of total in receipt of antidepressants*	30 (4.7)	25 (4.6)	30 (5.6)	85 (3.2)	170 (3.9)	*p* < 0.03

### Statistical analyses

Cox proportional hazards regression analysis was used to estimate hazard ratios for the risk of being prescribed antidepressant medicine. All analyses were carried out using SAS 9.4. In the main analyses, two models were calculated. The first model (Model 1a) included only procedural justice. In the second model (Model 2a), all of the covariates were added. Since depression is known to be a recurrent disease, and low procedural justice could have been present before study start, an argument could be made for including prior cases of antidepressant medication. Therefore, we decided to run sensitivity analyses including also participants with prior prescription of antidepressants (Model 1b and 2b, full sample).

To complement the main analyses and by testing accumulation of procedural justice as well as changes in these, additional analyses were performed using the summarized measure of procedural justice (sum score of procedural justice 2012 and 2014). For a detailed description, see Appendix 1 and Appendix 2. In these analyses, we ran the same models as described above, both on the restricted sample (excluding those who have had a prior prescription of antidepressant medication) and the full sample.

## Results

The 4374 individuals (1905 men, 2469 women) included had a mean age of 49.3 years in 2010 and the majority had completed an education of 3–4 years of upper secondary school or higher. Descriptive statistics for the four justice groups are shown in Table 1. Those experiencing stable low procedural justice were mainly women. The group of stable high levels of procedural justice consisted of relatively more men and people with an educational degree lower than university.

Table 2 presents hazard ratios for prescribed anti-depressives. We identified 170 events of new prescriptions of antidepressants when excluding any ongoing or prior events taking place in the 2 years up to answering SLOSH in 2012, i.e., approximately 4% of the total sample started treatment with antidepressants during follow-up. In the unadjusted model (Model 1a) including only procedural justice as explanatory variable, a significant overall effect of procedural justice was observed (*p* = 0.04). Specifically, compared to those with stable high justice, participants who perceived a decrease in procedural justice had a higher hazard of being prescribed antidepressants (HR 1.69, 95% CI: 1.12 to 2.56). No other significant differences were found, although both those reporting stable low (HR 1.40, 95% CI: 0.96 to 2.21) and increasing justice levels (HR 1.45, 95% CI: 0.92 to 2.24) showed slightly elevated hazard ratios. Inclusion of covariates (Model 2a) changed the results only marginally, those who perceived a decrease in procedural justice showed an elevated risk for antidepressant description. Specifically, the fact that there was a significant overall effect of procedural justice (*p* = 0.03) indicated that procedural justice had an effect on the hazard of being prescribed antidepressants. Female sex (HR 1.70, 95% CI: 1.22 to 2.36) and insecure employment in 2010 (HR 2.51, 95% CI 1.48 to 4.24) were significantly associated with a higher hazard of being prescribed antidepressant medicine. No other covariates predicted prescription of antidepressants in the final model.

**Table 2:** Cox regression models predicting antidepressant prescriptions by low procedural justice

Group	Model 1a Crude model	Model 2a* + Covariates	Model 1b° Crude model	Model 2b*° + Covariates
	*n* = 4374, 170 events		*n* = 4652, 448 events	
	HR (95% CI)	HR (95% CI)	HR (95% CI)	HR (95% CI)
Group 1: Stable low	1.46 (0.96 to 2.21)	1.46 (0.96 to 2.22)	**1.59 (1.24 to 2.03)**	**1.57 (1.22 to 2.01)**
Group 2: Increasing	1.44 (0.92 to 2.24)	1.47 (0.94 to 2.30)	**1.39 (1.05 to 1.83)**	**1.41 (1.07 to 1.86)**
Group 3: Decreasing	**1.77 (1.17 to 2.68)**	**1.79 (1.18 to 2.73)**	**1.65 (1.27 to 2.14)**	**1.67 (1.29 to 2.20)**

Notes: Reference group is group 4 (stable high)

*Models 2a and 2b are adjusted for sex, age, education, socioeconomic position, marital status, insecure employment in 2010 (estimates not shown, sex and insecure employment remained significant)

°Model 1b and 2b are adjusted for all covariates as above, and also includes events from individuals in receipt of prescribed antidepressant medication in the 2 years up to 2012

Sensitivity analyses were run including all participants regardless of prior prescription (*n* = 4652). In this sample, we identified 448 events, indicating that 9.9% of the sample received at least one prescription of antidepressant medication during follow-up. Results as provided in Table 2 (Models 1b and 2b) show that using the full sample, all groups, i.e., those with stable low, increasing and decreasing justice, had an increased hazard for prescribed antidepressants as compared to those reporting stable high procedural justice.

Results for accumulative effects showed only non-significant results using the restricted sample; however, all groups showed increased hazards for prescribed antidepressants using the full sample (see Appendix 1). Those with one standard deviation decrease in procedural justice showed a slightly increased hazard for prescription of antidepressants, which disappeared after controlling for the covariates (see Appendix 2).

## Discussion

This study investigated the longitudinal relationship between individual’s perception of procedural justice at two time points on the hazard of receiving prescribed antidepressant medication in a large cohort approximately representative of the Swedish working population. In line with our expectations, a decrease in perceptions of procedural justice over two measurement points was associated with an increased hazard of antidepressant medication prescription during follow-up. This result held stable also after the inclusion of relevant covariates. Interestingly, in the analyses based on the restricted sample (participants without prior antidepressant prescriptions), stable low perceptions of procedural justice were not related to a higher prevalence of new prescriptions of antidepressant medication. One possible explanation might be that the group was rather small (*n* = 170); thus power problems might at least partially explain the null-finding. Also, it is possible that justice perceptions influenced health already prior to the study, so that respondents had habituated to the situation, and that stable low justice was not related to a further decline in health [19, 38]. Indeed, analyses based on the full sample including those with prior prescription of antidepressants revealed an increased hazard also for participants experiencing stable low justice as well as increasing justice, suggesting that health problems and possibly low justice perceptions may have been present before inclusion in our study. This also indicates that low justice perceptions, at any time, may constitute a risk for mental ill-health, rather than a negative change per se. However, the changing groups do not change much over the period of 2 years with regards to their mean value, thus, it is important to stay cautious regarding whether it is change or rather low procedural justice that is driving the results.

To the best of our knowledge, our study is one of the first investigating procedural justice perceptions, along with changes in said perceptions over time, in relation to antidepressant medication. However, there are earlier studies investigating change in justice in relation to other outcomes. One such study was conducted by Kivimaki et al. (2004), using logistic regression analyses over three time points. They found that low and declining levels in interactional justice were related to a decrease in self-rated health [29]. Similarly, another study from Ferrie et al. (2006) found that an adverse change in interactional justice had an immediate and long-term risk on psychiatric morbidity [28]. Yet another recent study from Leineweber et al. (2016) demonstrated that changes in procedural justice were related to changes in self-rated health [30].

Our results finds support from both cross-sectional [8, 10, 11, 39, 40] and longitudinal [11, 19, 29, 30] studies investigating the relationship between procedural justice and depression. For example, one study by Ybema et al. (2010) with a longitudinal design reported that both distributive and procedural justice contributed to lower depressive symptoms the following year [11]. Yet another recent study from Eib et al. (2018) reported significant lagged effects from procedural justice to depressive symptoms [19]. However, while some have used non-subjective measures of depression, mostly defined as registry-based sickness absence [41] or disability pension [38] due to diagnosed mental disorder, many of the studies looking at organizational justice and health outcomes are exposed to the risk of common method variance. By using medication registers for the outcome, the present study avoided that risk.

Antidepressant medication is widely used in Sweden as first-line treatment in general practice for patients with symptoms of depressive disorders. Therefore, the Prescribed Drug Register has a very good coverage in general [42]. Although not a perfect measure of depression, since these medications may also be used to treat other illnesses, including anxiety disorders, chronic pain, sleeping problems, chronic fatigue, migraine, irritable bowel syndrome and social phobia, [43] it is likely that our outcome forms a clinical relevant outcome which covers serious mental ill-health.

The results that declining procedural justice is associated with subsequent antidepressant receipt provides support for a potential causal relationship from procedural justice to medication receipt. Specifically, using the conservative sample, only decreasing, but not stable low justice perceptions were related to increased hazard for prescribed antidepressants. Still, we obtained similar results regardless of sample used, with the difference that the conservative sample provided weaker results. However, reverse effects cannot be excluded; it is possible that those receiving antidepressant treatment had a more negative view of their work environment, including procedural justice. Such an idea finds support in one study by Lang et al. (2011), in which they found, based on three samples from different military contexts, only a relationship from depressive symptoms to subsequent organizational justice perceptions, while organizational justice perceptions showed no effect on subsequent depressive symptoms [44]. Another study report both causal and reversed relationships between procedural justice perceptions and depressive symptoms [19].

We also found that women as well as those in insecure employments showed an increased risk for antidepressant receipt. The latter is an interesting finding, and similar results in relation to register data of antidepressant use has been reported elsewhere [45]. One possible mechanism could be that an insecure employment implies low employability, which may, in turn, affect one’s justice perception [46]. Another potential mechanism could be that an insecure employment breaches the psychological contract [47]. More research is needed to examine the relation between an insecure employment and its influence on justice perceptions, as well as the effects of other organizational changes on both justice perceptions and prescriptions of antidepressant medication.

### Strengths and limitations

This study has several strengths. It uses data that are close to representative of the Swedish in-work population. Analyses were conducted prospectively for the association between changes in perceptions of procedural justice, using a validated scale with good measurement properties, and receipt of antidepressant medication. The number of individuals with a prescription for antidepressant medications in the restricted sample was 170 (about 4%), while in the full sample that number was 448 (about 10%), which is comparable to the Swedish nationwide statistics of 8.1% of the population having a prescription of antidepressant medications [48]. In using a prescription register to record the outcome, rather than using self-reports for both exposure and outcome, the results are not vulnerable to common-method bias, a frequent difficulty in prior research in this area. However, this study also has certain limitations.

First, the use of receipt of antidepressant medication is not a perfect measure of depressive disorders and has likely generated some misclassification of the outcome. Medication from the N06A category is not only prescribed for depression and anxiety disorders, but also for insomnia, eating disorders, migraine and so forth [43]. In addition to mental ill-health, the receipt of antidepressant medication measures a social process of seeking medical advice, gaining an appointment and being prescribed and purchasing a pharmaceutical from the N06A category. While this is problematic, it is still a relevant health indicator. Given that a sensitivity analysis including self-reported depression rendered all results null, the prescribed medication does arguable indicate mental health issues. Also, two prior studies gives support to the claim that antidepressants can be used as a proxy for depressive disorders in a Swedish setting [32, 49]. This is in line with information from the National Health Service in the UK who states that ‘the main use for antidepressants is treating clinical depression in adults’. [50] Secondly, attrition analyses revealed that participants reporting major depression in 2010, were more likely to answer to the non-worker questionnaire in 2012 and slightly more likely not to answer at all. This indicates that our data may be biased towards healthy workers, especially drop-out among those with high levels of depression in 2010 might underestimate any true effect. Third, we only looked at the exposure to procedural justice, while ignoring other parts of the organizational justice framework as well as other psychosocial working conditions known to affect health [12]. It is possible that other psychosocial working conditions have played a mediating or confounding role. However, we do not argue for procedural justice being the single most important working condition in relation to employee health, only one part of a bigger picture. Finally, another potential issue has to do with the way we created the four groups. The quartile split we implemented has the disadvantage that it is a rough estimate and that it does not take into consideration the amount of individual differences over time. However, alternative methods like sum scores or changes in standard deviation over time also come at a cost. When running our additional analyses on the groups looking at accumulated effects over time, using the alternative methods of sum score and standard deviation change measuring the exposure, the results were similar to the ones presented in our main analyses (see Appendix 1 and 2).

## Conclusion

In conclusion, the current study improves the evidence base of the relationship between organizational justice and mental health by investigating associations between self-reports of procedural justice and register data on receipt of antidepressant medication. We found that a decrease in perceived procedural justice was associated with a higher risk of being prescribed antidepressants. These findings contribute by confirming and building on earlier research on the effects of procedural justice at the workplace and its link to mental ill health and depression, with the use of a health outcome obtained from register data rather than from self-reports. Further longitudinal research is needed to better assess which work-related concepts predict mental health, as well as more research that look into how well antidepressant medication correlates with depressive symptoms.

## Data Availability

The datasets generated and/or analyzed during the current study are not publicly available due to legal restrictions but are available from the corresponding author on reasonable request.

## Appendix 1

### Cox regression sensitivity analyses predicting antidepressant prescriptions by low procedural justice

To further assess whether accumulation of perceived justice over time had an effect on depression medication, a sum score was calculated for each year of 2010–2012, thus creating four groups: *‘Lowest’* (summed score of 14 to 28), *‘Low’* (summed score of 29 to 42), *‘High’* (summed score of 43 to 56) and *‘Highest*’ (summed score of 57 to 70).

**Table:**

Group	Model 3a Crude model	Model 4a* +Covariates	Model 3b Crude model	Model 4b*° +Covariates
	*n* = 4374, 170 events		*n* = 4652, 448 events	
Sum score	HR (95% CI)	HR (95% CI)	HR (95% CI)	HR (95% CI)
Group 1: Lowest (14–28)	1.63 (0.82 to 3.25)	1.56 (0.78 to 3.11)	**2.78 (1.88 to 4.10)**	**2.68 (1.82 to 3.97)**
Group 2: Low (29–42)	1.38 (0.86 to 2.20)	1.37 (0.86 to 2.20)	**1.72 (1.26 to 2.33)**	**1.69 (1.24 to 2.30)**
Group 3: High (43–56)	1.25 (0.80 to 1.97)	1.23 (0.78 to 1.94)	**1.40 (1.04 to 1.89)**	**1.38 (1.02 to 1.86)**

Notes: Model 3a-3b and 4a-4b reference group is *‘Highest’* (57–70) group

*Models 4a and 4b are adjusted for sex, age, education, socioeconomic position, marital status, insecure employment in 2010 (estimates not shown, sex and insecure employment remained significant)

°Model 3b and 4b are adjusted for all covariates as above, and also includes events from individuals in receipt of prescribed antidepressant medication in the 2 years up to 2012.

## Appendix 2

### Cox regression sensitivity analyses predicting antidepressant prescriptions by change in standard deviation of procedural justice

We also wanted to assess whether other measures of change in the exposure had an effect on the outcome. Therefore, we looked at a standard deviation change in the perception of justice between 2010 and 2012. If there was an increase of 1 SD or more, a person was categorized into the ‘*Increasing’* group, while a person with a decrease of 1 SD or more, was categorized as ‘*Decreasing’*. If there was no change either up or down that equaled 1 SD or more, one was categorized as *‘Stable’*.

**Table:**

Group	Model 5a Crude model	Model 6a* +Covariates	Model 5b° Crude model	Model 6b*° +Covariates
	*n* = 4374, 170 events		*n* = 4652, 448 events	
SD-change	HR (95% CI)	HR (95% CI)	HR (95% CI)	HR (95% CI)
Group 1: Decreasing	**1.55 (1.03 to 2.33)**	1.45 (0.96 to 2.18)	**1.30 (1.00 to 1.69)**	1.24 (0.96 to 1.62)
Group 2: Increasing	1.45 (0.95 to 2.20)	1.43 (0.94 to 2.18)	1.21 (0.92 to 1.58)	1.20 (0.91 to 1.57)

Notes: Model 5a-5b and 6a-6b reference groups is *‘No change’* group

*Models 6a and 6b are adjusted for sex, age, education, socioeconomic position, marital status, insecure employment in 2010 (estimates not shown, sex and insecure employment remained significant)

°Model 5b and 6b are adjusted for all covariates as above, and also includes events from individuals in receipt of prescribed antidepressant medication in the 2 years up to 2012.

## Abbreviations

SLOSH: Swedish Longitudinal Survey of Health
WHO: World Health Organization
SWES: Swedish Work Environment Surveys
